# Technology-aided community engagement for exercise promotion: a mixed-methods systematic review

**DOI:** 10.1093/eurpub/ckac131.182

**Published:** 2022-10-25

**Authors:** GM Raspolini, MT Riccardi, G Damiani

**Affiliations:** 1 Igiene e Medicina Preventiva, Università Cattolica del Sacro Cuore, Rome, Italy; 2 Igiene e Medicina Preventiva, Fondazione Policlinico Universitario Agostino Gemelli IRCCS, Rome, Italy

## Abstract

**Background:**

This review aims to provide an overview of evidence on feasibility and effectiveness in diverse populations of eHealth physical activity (PA) community engagement (CE) interventions. Increasing global PA levels would have a substantial positive impact on population health. Given their diffusion, eHealth technologies may address certain barriers to PA and reach wide audiences. The most recent Italian guidelines on PA highlight inequalities in health, which can be addressed using CE models. The potential scalability of successful eHealth CE interventions and the scarcity of previous reviews on the topic are reasons which convinced us to work on this paper.

**Methods:**

This mixed-methods systematic review utilized the Joanna Briggs Institute methodologies. Primary quantitative outcome measures were minutes of PA per week. Qualitative outcome measures included self-efficacy and user engagement. Data were processed using a segregated convergent design. A narrative summary and a meta-aggregation were performed for synthesizing quantitative and qualitative data respectively. Only the interventions where CE principles were fulfilled were analyzed.

**Results:**

Quantitative evidence supported effectiveness and feasibility of interventions to improve PA outcomes and related proxy indicators across studied populations. Qualitative findings suggest the utility of peer-support and that from other health care providers.

**Conclusions:**

Implementing CE in future PA interventions will be critical for producing an effective digital application with the potential for considerable impact in the real world. If supported by central governments and the European Union, entities such as primary care hubs and local health units with their professionals and CE capabilities may play the key role in implementing evidence.

**Key messages:**

• eHealth PA CE interventions work better when peer support takes place.

• Health systems could pursue these prevention strategies for population health gains.

